# Adaptation to university life, fear of loneliness, and depression in first-year students at a private university in the Peruvian Amazon: a structural equation model

**DOI:** 10.3389/fpsyg.2026.1935089

**Published:** 2026-10-14

**Authors:** Rosa Gebol-Checàn, Gianella Ascorbe-Peña, Royer Miguel-Mariño, Calixto Tapullima-Mori, Julio Cjuno, Jhordan Mayta-Llanos

**Affiliations:** 1 Escuela Profesional de Psicología, Universidad Peruana Unión, Tarapoto, Peru; 2 Unidad de Posgrado de Psicología, Universidad Peruana Unión, Lima, Peru

**Keywords:** depression, fear of loneliness, first-year students, Peruvian students, university adaptation

## Abstract

**Introduction:**

The transition to university is a period of heightened psychological vulnerability. This study aimed to examine the association between adaptation to university life, fear of loneliness, and depression in Peruvian first-year university students.

**Methods:**

A quantitative cross-sectional study was conducted with 345 participants selected through non-probability convenience sampling at a single private university in the Peruvian Amazon. The Student Adaptation to College Questionnaire, the Brief Scale of Fear of Loneliness, and the Patient Health Questionnaire-9 were administered, and data were analyzed using structural equation modeling (SEM).

**Results:**

The proposed model showed an adequate fit [*χ*² (516) = 1,478, *p* < 0.001, CFI = 0.940, RMSEA = 0.074, SRMR = 0.069]. Personal-emotional adjustment (*β* = 0.49, *p* < 0.001) and social adjustment (β = 0.29, *p* = 0.003) were positively associated with fear of loneliness, whereas academic adjustment (*β* = −0.34, *p* < 0.001) and institutional adjustment (*β* = −0.22, *p* = 0.001) were negatively associated; in turn, fear of loneliness was positively related to depression (β = 0.81, *p* < 0.001). Significant gender differences were found in institutional adjustment [*t* (343) = 2.597, *p* = 0.010, *d* = 0.29] and personal-emotional adjustment [*t* (343) = 3.076, *p* = 0.002, *d* = 0.34], with women reporting higher levels than men.

**Discussion:**

The findings position fear of loneliness as a key affective link between first-year students’ adjustment to university and their depressive symptoms, suggesting that early detection and intervention programs focused on adjustment, implemented during the first year, could help mitigate depression in this population.

## 1 Introduction

The transition to higher education is a significant challenge for first-year students, as they face multiple obstacles that can affect their academic performance and general well-being (Gosai et al., 2023). Approximately one third of university students experience adaptation difficulties when beginning their studies (Samašonok et al., 2025). These difficulties can be classified into external and internal factors: on the one hand, students are overwhelmed by the lack of clear instructions for assigned tasks, the considerable amount of academic activities, the intensity of the modular system, and the excessive workload and assessments; on the other hand, internal factors involve psychological and emotional demands, such as difficulties with self-regulation, emotion management, and coping (Li and Lee, 2025; Lubis et al., 2025).

International studies document that 81.5% of university students report complications associated with depression, with physical exercise and psychopathological history as significant predictors (Malebari et al., 2024). Among South Asian university students, the presence of Major Depressive Disorder was 31%, and 70% reported having experienced some type of depression ranging from mild to severe (Wickramasinghe et al., 2023). In Peru, 47.8% of university students presented depression problems, which confirms the magnitude of the problem (Moya et al., 2025).

It is well known that first-year university students are exposed to significant stressors that hinder their adaptation; socioemotional challenges, economic hardship, and coping difficulties are components that generally tend to have a negative impact on their settling into this new stage (Aderi et al., 2013; Li and Lee, 2025). A study in Ethiopia showed that university adaptation problems reached 41.1%; among these, factors such as the distance between home and university, money to support oneself, and living away from home and family for the first time were aspects associated with greater adaptation problems (Anbesaw et al., 2022).

On the other hand, it has been identified that the problem of loneliness is becoming a common issue among university students, which erodes mental health and predisposes them to psychological complications such as depression (Xie et al., 2025); as shown by a study from Iran that reported that 42.42% of students felt lonely (Zahedi et al., 2022). In the United States, it was corroborated that university students had high levels of self-reported loneliness, reaching up to 41%. First-year students had a rate of 18.5% when reporting that there was no one to turn to for help, and 25.9% showed a tendency to fear loneliness (Wodika et al., 2024).

In Peru, first-year university students present moderate and low adaptation problems at 57 and 17.9%, respectively; moderate levels stand out, such as personal adaptation at 51.6%, adaptation to the career at 51.1%, and adaptation to study at 59.5% (Tataje et al., 2024). Little scientific evidence was found showing the prevalence or percentages of university students with fear-of-loneliness problems, especially among those in their first year; however, it was found that 3%, 2%, and 8% of them had depression problems at extremely severe, severe, and moderate levels (Mejia et al., 2022).

This study is based on the Student Integration Model, which describes the processes and factors that lead a student to adapt to the academic context, remain in it, and thereby avoid student dropout (Tinto, 2012). The key component is the integration and patterns of interconnection among the members of the educational community and the social interaction of students, which serve as a protective factor against the psychological distress resulting from academic workload (Rodríguez et al., 2017).

A second foundation for the soundness of the theoretical model is the Cognitive Discrepancy Theory, which posits that people experience loneliness when they perceive that their desired and actual levels of social interaction are not aligned (Perlman and Peplau, 1982). This discrepancy, over time, generates distress in people that can be reflected in psychological problems such as indicators of anxiety and depression, which erodes psychological health and affects the academic domain of students (Archibald et al., 1995).

Taken together, these two frameworks provide an integrated rationale for the proposed model rather than a set of isolated paths: the Student Integration Model (Tinto, 2012) explains how the academic, institutional, and social dimensions of adaptation shape students’ connection with the university and, thereby, their vulnerability to socio-affective distress such as fear of loneliness, whereas the Cognitive Discrepancy Theory (Perlman and Peplau, 1982) accounts for how this distress is, in turn, associated with depressive symptomatology. Each hypothesized relationship therefore derives from a specific theoretical premise, and the model as a whole reflects the joint contribution of both frameworks.

### 1.1 Adaptation to university life and fear of loneliness

It has been documented that the transition to the university stage presents risks of loneliness and mental health problems; specifically, levels of social capital, satisfaction with the university induction, and sense of belonging were significantly associated with lower levels of loneliness (Thomas et al., 2020). Another study conducted in Iran reported that first-year university students presented the highest rates of loneliness compared to their peers in higher years (Zahedi et al., 2022), thus confirming the literature holding that interpersonal relationships and connection with the university can help mitigate the effects of loneliness (Wenig et al., 2025), while at the same time promoting greater academic engagement (Padilla-Guevara et al., 2026). That study drew on an independent sample and addressed academic engagement; the present research instead tests how adaptation to university life relates to fear of loneliness and, in turn, to depression—an anticipatory-fear pathway, distinct from experienced loneliness, not yet examined in this population.

### 1.2 Fear of loneliness and depression

Although the variable of interest in the present study is fear of loneliness, it is necessary to distinguish it conceptually from loneliness. Loneliness is a perceived state that arises from inadequate satisfaction of interpersonal relationships (Andersson, 1993); fear of loneliness constitutes an anticipatory and avoidant disposition, characterized by concern about a possible social disconnection (Wolters et al., 2023). This distinction is theoretically relevant, since anticipatory fear may operate differently from experienced loneliness, by acting upon cognitive and motivational processes even before social isolation occurs (Ventura et al., 2020). Therefore, and given the scarcity of literature associating fear of loneliness with depression, the available evidence on the relationship between loneliness and depression offers a pertinent frame of reference to support the research hypothesis.

In light of the above, a study conducted in Oman showed that there is a direct and positive relationship between the two variables, since the feeling of loneliness tends to invalidate the person, and therefore depression problems become more frequent; moreover, 43% of the variance in depression was explained by loneliness (Abu et al., 2023). In Ireland, it was shown that loneliness significantly increases the probability of depression by up to 48% in the university population (Qirtas et al., 2023). Along the same lines, a study carried out in Vietnam revealed that university students have a higher risk of experiencing loneliness and depression as interconnected psychological conditions (Pham et al., 2024). Regarding the Peruvian context, only one study was found that associated loneliness with suicidal ideation, but mediated through depression; that is, the latter condition lies within the relationship between loneliness and depressive symptoms (de la Cruz et al., 2023).

Based on the above, the rationale for this research lies in the lack of an explanatory model in the scientific literature involving a Peruvian university population that examines the associations between student adaptation, fear of loneliness, and depression among first-year university students. The results are intended not only to provide an integrated theoretical framework for understanding the study variables and their functioning, but also to serve in the formulation of future treatment programs for students who are integrating into university life.

Regarding fear of loneliness, being a unidimensional construct whose items assess fear of abandonment and concern about being alone, it plays a dual role in the explanatory model: it acts as a criterion variable with respect to the dimensions of university adjustment, and as a predictor variable of depression. This distinction is pertinent because the dimensions of adaptation to university life assess differentiated psychological processes such as personal-emotional aspects, academic competence, institutional commitment, and social integration, whose relationship with fear of loneliness operates through distinct pathways and should not be collapsed into a composite score (Dominguez et al., 2021). In line with the Cognitive Discrepancy Theory (Perlman and Peplau, 1982), the discrepancy between desired and actual levels of social interaction generates psychological distress that may manifest as depressive symptomatology (Archibald et al., 1995).

### 1.3 Objectives and hypotheses

The general objective of this study was to determine the relationship between the dimensions of adaptation to university life, fear of loneliness, and depression in first-year students at a private university in the Peruvian Amazon. From this aim, five specific objectives were derived, each formulated as a directional hypothesis (H1–H5). The conceptual model is shown in Figure 1.

**Figure 1:**
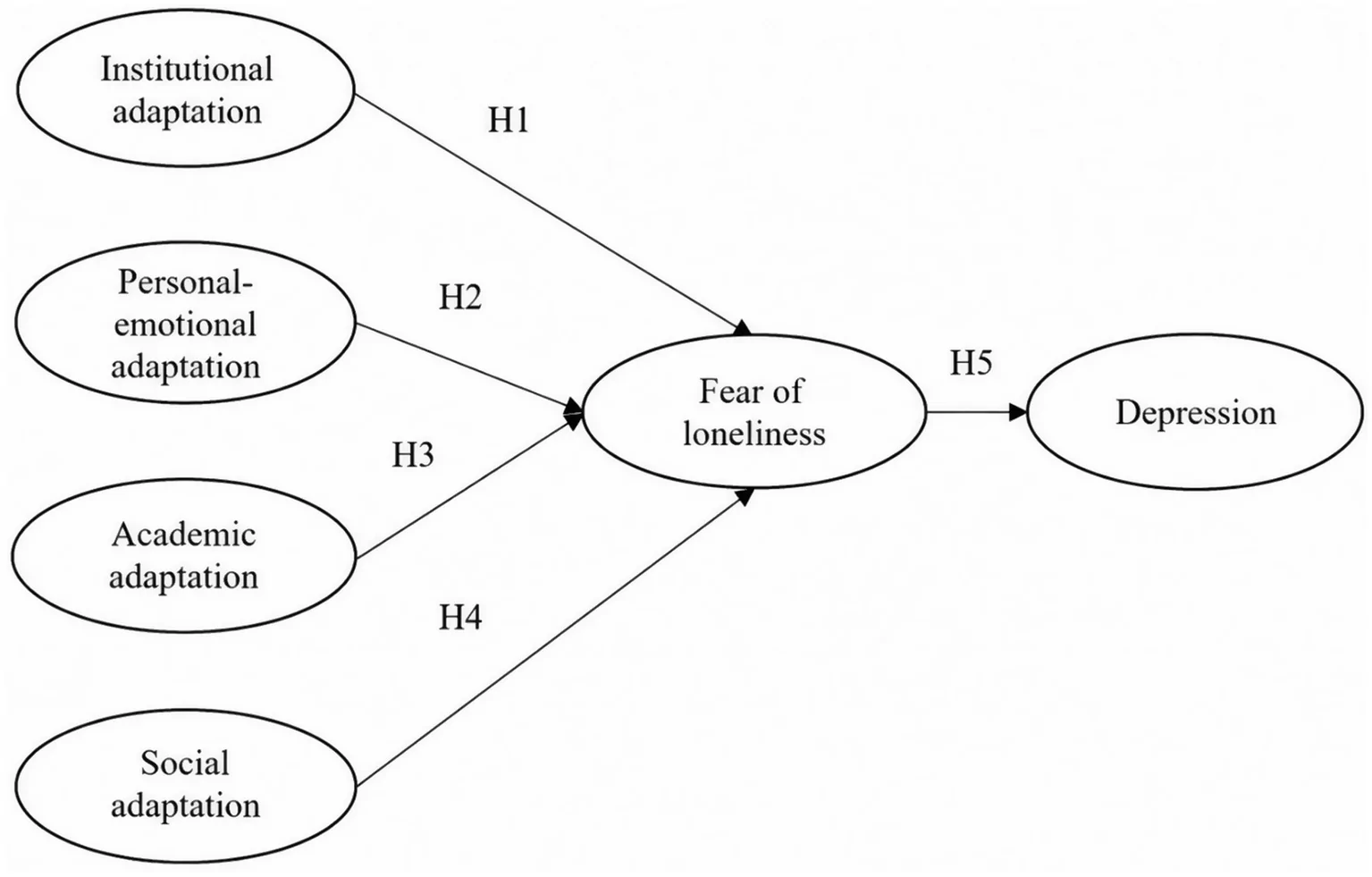
Theoretical model.

H1. There is a significant negative relationship between institutional adjustment and fear of loneliness in Peruvian first-year university students.

H2. There is a significant positive relationship between personal-emotional adjustment and fear of loneliness in Peruvian first-year university students.

H3. There is a significant negative relationship between academic adjustment and fear of loneliness in Peruvian first-year university students.

H4. There is a significant negative relationship between social adjustment and fear of loneliness in Peruvian first-year university students.

H5. There is a significant positive relationship between fear of loneliness and depression in Peruvian first-year university students.

## 2 Materials and methods

### 2.1 Design

The present research used a quantitative, non-experimental, cross-sectional design with an associative and explanatory scope (Ato et al., 2013). Variables were measured at a single point in time and without manipulation; this design is appropriate for testing a model of hypothesized relationships among latent variables through structural equation modeling.

A non-experimental design was adopted because the constructs of interest—adaptation to university life, fear of loneliness, and depression—are psychological states that cannot be ethically or practically manipulated, and whose meaning depends on students’ authentic experience in the transition to higher education. The study’s objective was explanatory, not causal: to test whether a theoretically derived pattern of associations among these constructs holds true in a sample of first-year Peruvian students. When a cross-sectional design is combined with a pre-specified model and error-corrected latent variable estimation, it can provide significant evidence of hypothesized relationships and help rule out several alternative explanations; therefore, experimental manipulation was neither feasible nor necessary to achieve this objective (Spector, 2019).

### 2.2 Participants

The sample was determined using the calculator Soper (2026) applying Monte Carlo simulation techniques, following the criteria established by Wolf et al. (2013) for Structural Equation Modeling (SEM) studies. The specified parameters included an anticipated effect size of 0.3, a desired statistical power of 0.8, a probability level of 0.05, and a structural model composed of 6 latent variables and 34 observed variables; this analysis determined an acceptable recommendation of 161 participants to ensure the statistical adequacy of the SEM analysis. Nevertheless, 345 students were surveyed, far exceeding this threshold and ensuring the robustness of the analyses conducted. The participant selection process, including the application of the exclusion criteria, is presented in Figure 2.

**Figure 2:**
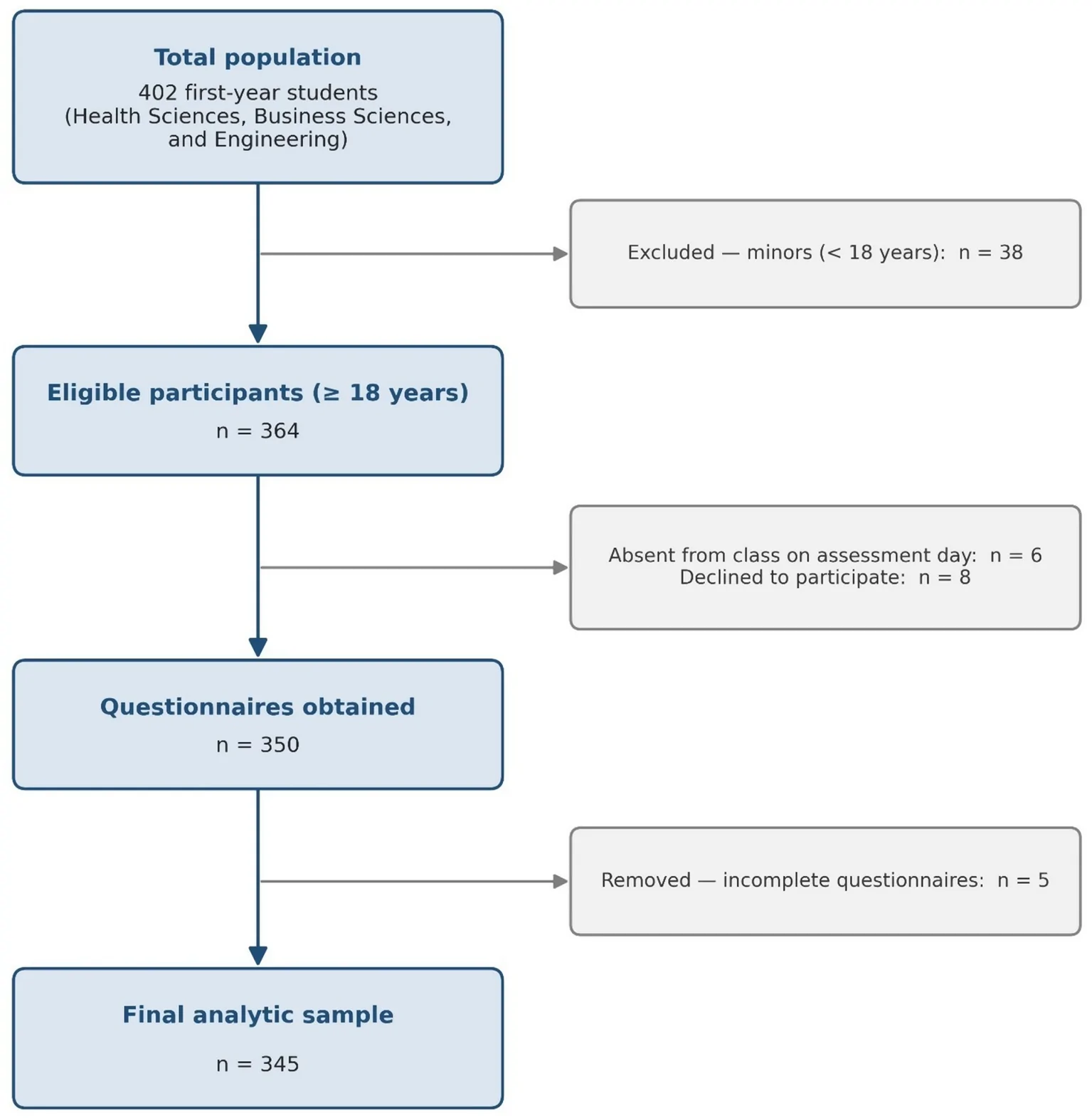
Flow diagram of the participant selection process.

Table 1 shows the distribution of the participants, where age had a mean of 18.8 and a standard deviation of 2.06; the majority were female (61.7%), belonging to the Adventist religious denomination (40.3%), and were studying careers associated with Health Sciences (40.3%).

**Table 1:** Sociodemographic data of the participants.

Variable	Characteristic	Frequency	%
Sex	Female	213	61.7
	Male	132	38.3
Religious denomination	Adventist	139	40.3
	Catholic	120	34.8
	Evangelical	32	9.3
	Other	54	15.7
Professional field to which your degree belongs	Health sciences	139	40.3
	Social sciences, business and law	73	21.2
	Education	43	12.5
	Engineering, manufacturing and construction	90	26.1

### 2.3 Instruments

The Student Adaptation to College Questionnaire (SACQ) was initially created by Baker and Siryk (1989) and validated for the Peruvian context by Dominguez et al. (2021); it comprises a total of 27 items. It has a Likert-type response format where 1 (strongly disagree), 2 (does not relate to me), 3 (rarely happens), 4 (at other times disagree), 5 (sometimes), 6 (at other times agree), 7 (happens quite often), 8 (very much relates to me), and 9 (strongly agree). Likewise, it presented adequate evidence of reliability for the dimensions of institutional adjustment (*ω* = 0.839), personal-emotional adjustment (*ω* = 0.863), academic adjustment (*ω* = 0.687), and social adjustment (*ω* = 0.751), as well as adequate fit indices (RMSEA = 0.044; CFI = 0.979). It should be noted that this instrument has two reverse items (5 and 6), so these will be scored in reverse during data coding.

The Brief Scale of Fear of Loneliness (BSFL) was originally created by Ventura et al. (2020), which is composed of 5 items in a unidimensional format with 5 response options: 0 (never), 1 (rarely), 2 (sometimes), 3 (almost always), and 4 (always). It has an adequate internal structure reflected in its omega value (*ω* = 0.91), as well as adequate fit indices (*χ*^2^ = 12.93, df = 5, *χ*^2^/df = 2.59, RMSEA = 0.04, CFI = 1.00, TLI = 1.00).

The Patient Health Questionnaire (PHQ-9) was originally developed by Kroenke et al. (2001), and adapted for the Peruvian context by Huarcaya et al. (2020); it has a total of 9 items presented in a bifactorial format. The scale has 4 Likert-type response options: 0 (not at all), 1 (several days), 2 (more than half the days), and 3 (nearly every day). The instrument showed good internal consistency for its two factors—somatic and cognitive/affective—and as a general factor (*α* = 0.903), as well as good model fit [*χ*^2^ (17) = 26.451, *p* = 0.067; CFI = 0.991; GFI = 0.969; RMSEA = 0.056].

### 2.4 Procedure

Data collection was carried out considering the guidelines of the Declaration of Helsinki, which ensures that ethical and data protection standards are met. Likewise, an online form was created using the Microsoft 365 Forms platform, in which the first section included informed consent, thereby preserving voluntary participation; the objective of the research and the procedure were also explained, emphasizing confidentiality, anonymity, the exclusive use of information for academic purposes, and the appropriate disposal of the acquired data, taking into account the ethics for their proper handling. The second section of the form included the collection of sociodemographic data, followed by the section with the research questionnaires.

All three instruments had been previously validated for the Peruvian context; therefore, no additional validation was conducted in the present study, and the psychometric properties reported for each scale—together with the reliability obtained in the present sample—are summarized above.

### 2.5 Data analysis

Data were processed in Microsoft Excel (variable coding, reverse-scoring of institutional-adjustment items 5 and 6, computation of dimension and total scores, and removal of incomplete cases) and analyzed in Jamovi 2.6.44 (The Jamovi Project, 2023), with the structural equation model estimated through the lavaan package in R (Rosseel, 2012).

In the measurement model, adaptation to university life was specified as four correlated first-order factors (institutional, personal-emotional, academic, and social adjustment) at the item level; fear of loneliness as a unidimensional factor; and depression as a second-order factor over its somatic and cognitive-affective subscales, consistent with the PHQ-9 structure (Huarcaya et al., 2020). Combining item-level indicators and subscale scores within a single measurement model is an accepted practice supported by the parceling literature (Little et al., 2002, 2013). Because all indicators were ordinal, the model was estimated with the WLSMV estimator, which does not assume multivariate normality and yields less biased estimates than maximum likelihood with categorical data (Lei and Wu, 2012; Li, 2016; Yildrim et al., 2018), and which performs adequately with ordinal indicators and moderate sample sizes (Holtmann et al., 2016; Lei and Shiverdecker, 2020).

The personal-emotional subscale is worded in terms of distress and is not reverse-scored, so higher scores denote greater difficulty and its coefficients are interpreted accordingly (Dominguez et al., 2021). Model fit was evaluated with RMSEA < 0.08, SRMR < 0.08, and CFI > 0.90 (Hooper et al., 2008); a hypothesis was considered supported when its standardized structural coefficient was statistically significant (*p* < 0.05) and in the predicted direction. Internal consistency was assessed with McDonald’s omega (*ω*) at the global and dimension levels. Given the cross-sectional design and the estimation of first-order direct paths only, all relationships are interpreted as associations rather than causal or mediational effects.

### 2.6 Ethical aspects

The principles of autonomy, beneficence, and justice guided the research process, ensuring respect for the participants’ will, the promotion of benefits, and the reduction of risks, as well as a transparent selection and handling of data. Ethical principles were crucial to guarantee transparency, responsibility, and integrity throughout the research (Miranda and Villasís, 2019). It should be noted that the corresponding permission was also requested from the research ethics and bioethics committee of the Faculty of Health Sciences of the Universidad Peruana Unión, under number 2025-CEB-FCS-UPeU-188.

## 3 Results

### 3.1 Descriptive analysis of the variables

In Table 2, the skewness values are within the ±3 range, indicating that they are within acceptable limits (Kline, 2023); likewise, the results for the *ω* were above 0.70, thus reflecting adequate internal consistency of the instruments (Cheung et al., 2024).

**Table 2:** Descriptive statistics of the variables and their dimensions.

Variables	M	SD	*g*_1_	*ω*
Institutional adjustment	35.2	6.8	−2.431	0.73
Personal-emotional adjustment	60.6	24.2	0.288	0.94
Academic adjustment	26.3	7.5	−0.760	0.87
Social adjustment	25.8	8.4	−0.598	0.88
Fear of loneliness	6.1	4.9	0.634	0.91
Somatic	3.5	2.4	0.428	0.72
Cognitive/affective	5.9	4.7	0.586	0.90
Depression	9.4	6.6	0.481	0.91

M, mean; SD, standard deviation; *g*_1_, skewness; *ω*, McDonald’s Omega.

### 3.2 Mean comparison analysis

An independent-samples Student’s *t*-test was conducted to examine possible gender differences in the six study variables. The results revealed statistically significant differences in Institutional Adjustment, with women (M = 35.89, SD = 5.62) reporting higher scores than men (M = 33.95, SD = 8.28), *t* (343) = 2.597, *p* = 0.010, with a small effect size (*d* = 0.29). Similarly, Personal-Emotional Adjustment was significantly higher in women (M = 63.69, SD = 23.74) than in men (M = 55.53, SD = 24.29), *t* (343) = 3.076, *p* = 0.002, with a small effect size (*d* = 0.34). In contrast, no significant gender differences were observed for Academic Adjustment [*t* (343) = 1.422, *p* = 0.156], Social Adjustment [*t* (343) = 0.539, *p* = 0.591], Fear of Loneliness [*t* (343) = 0.519, *p* = 0.604], or Depression [*t* (343) = 1.952, *p* = 0.052]. The effect sizes for the non-significant comparisons ranged from negligible to small (*d* = 0.06–0.22) (see Table 3).

**Table 3:** Mean comparison of the study variables by gender.

Variable	Gender	M	SD	*t*	df	*p*	Cohen’s *d*
Institutional adjustment	Female	35.9	5.6	2.60*	343	0.010	0.29
	Male	34.0	8.3				
Personal-emotional adjustment	Female	63.7	23.7	3.08*	343	0.002	0.34
	Male	55.5	24.3				
Academic adjustment	Female	26.7	7.1	1.42	343	0.156	0.16
	Male	25.6	8.1				
Social adjustment	Female	26.0	7.8	0.54	343	0.591	0.06
	Male	25.5	9.3				
Fear of loneliness	Female	6.2	4.8	0.52	343	0.604	0.06
	Male	5.9	4.9				
Depression	Female	9.9	7.0	1.95	343	0.052	0.22
	Male	8.5	5.9				

M, mean; SD, standard deviation; *t*, Student’s *t*; df, degrees of freedom; *p*, significance. *p < 0.05.

### 3.3 Correlation analysis

Table 4 shows the correlation analysis, which revealed that personal-emotional adjustment was the only dimension positively and significantly associated with fear of loneliness, while institutional, academic, and social adjustment did not show a significant relationship with this variable. Furthermore, fear of loneliness was positively and significantly associated with depression, suggesting that students who experience greater fear of being alone tend to exhibit higher levels of depressive symptoms.

**Table 4:** Correlation analysis among the variables.

Variable	1	2	3	4	5	6	7	8
1. Institutional adjustment	—							
2. Personal-emotional adjustment	0.17^**^	—						
3. Academic adjustment	0.45^***^	0.15^**^	—					
4. Social adjustment	0.46^***^	0.08	0.67^***^	—				
5. Fear of loneliness	−0.03	0.37^***^	−0.08	−0.02	—			
6. Somatic	0.03	0.44^***^	−0.08	−0.07	0.45^***^	—		
7. Cognitive	−0.06	0.51^***^	−0.17^**^	−0.17^**^	0.49^***^	0.72^***^	—	
8. Depression	−0.03	0.52^***^	−0.15^**^	−0.14^**^	0.51^***^	0.87^***^	0.97^***^	—

^*^p < 0.05; ^**^p < 0.01; ^***^p < 0.001.

### 3.4 Structural model analysis

The fit of the SEM model was adequate: *χ*^2^(516) = 1,478; *p* < 0.001; *χ*^2^/df = 2.86; CFI = 0.940; RMSEA = 0.074; SRMR = 0.069. It should be noted that Figure 3 constitutes a simplified representation of the complete model for presentation purposes; nevertheless, all paths, covariances, and coefficients were estimated including all the items of each dimension (Supplementary Figure A1).

**Figure 3:**
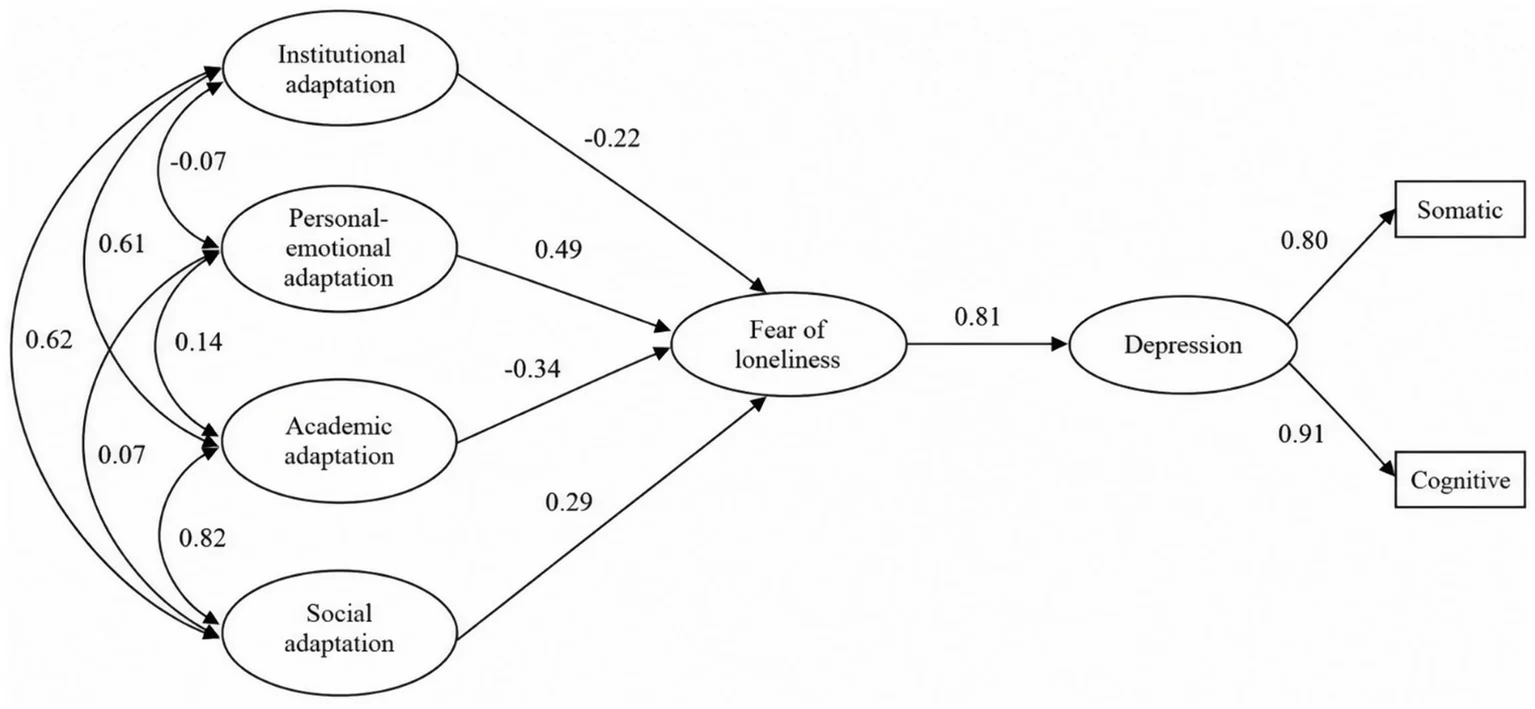
Statistical model.

Regarding the correlates of fear of loneliness, personal-emotional adjustment showed the strongest positive association (*β* = 0.49, *p* < 0.001), whereas academic adjustment (*β* = −0.34, *p* < 0.001) and institutional adjustment (*β* = −0.22, *p* = 0.001) showed significant negative relationships, representing inverse associations. Social adjustment was positively associated with fear of loneliness (*β* = 0.29, *p* = 0.003). Together, the adjustment dimensions explained 32.7% of the variance in fear of loneliness. In turn, fear of loneliness was positively related to depression (*β* = 0.81, *p* < 0.001), explaining 65.0% of its variance.

In hypothesis-testing terms, H1, H2, H3, and H5 were supported, whereas H4 was not supported: social adjustment had been hypothesized to relate negatively to fear of loneliness, but its structural coefficient was positive (*β* = 0.29, *p* = 0.003). Because these structural coefficients are affected by suppression, they should not be interpreted as zero-order associations (cf. Table 4); a summary of all hypothesis decisions is provided in Table 5.

**Table 5:** Summary of the tested hypotheses and decisions.

Hypothesis	Structural path	Hypothesized direction	*β*	*p*	Decision
H1	Institutional adjustment → fear of loneliness	Negative	−0.22	0.001	Supported
H2	Personal-emotional adjustment → fear of loneliness	Positive	0.49	<0.001	Supported
H3	Academic adjustment → fear of loneliness	Negative	−0.34	<0.001	Supported
H4	Social adjustment → fear of loneliness	Negative	0.29	0.003	Not supported (direction reversed)
H5	Fear of loneliness → depression	Positive	0.81	<0.001	Supported

*β* = standardized structural coefficient. Coefficients derive from the SEM and may differ from the zero-order correlations in Table 4 owing to suppression effects.

It should be noted that these structural coefficients differ from the bivariate correlations reported in Table 4, in which institutional, academic, and social adjustment showed no significant associations with fear of loneliness. This discrepancy is consistent with a statistical suppression effect, widely documented in the psychometric literature (Cohen and Cohen, 1975; Paulhus et al., 2004), which occurs when highly intercorrelated predictors—in this case, the four adjustment dimensions, with intercorrelations ranging from 0.15 to 0.67—are entered simultaneously into a multivariate model: by controlling for the shared variance among them, the unique relationship of each predictor with the criterion variable emerges, which may differ in magnitude and even in direction with respect to its isolated bivariate association (Watson et al., 2013).

### 3.5 Discriminant validity and measurement model evaluation

Discriminant validity between fear of loneliness and depression was supported: the HTMT ratio was 0.58, below the 0.85 threshold, and the average variance extracted by each construct (0.72 and 0.79) exceeded their shared variance (*r*^2^ = 0.26), meeting the Fornell and Larcker (1981) criterion (Table A4); the two scales also share no items, as the BSFL assesses anticipatory apprehension about being alone and the PHQ-9 assesses current depressive symptoms. The higher structural coefficient between the two latent variables (*β* = 0.81; Table A2) relative to the correlation between their observed scores (*r* = 0.51) is attributable to disattenuation—latent paths are corrected for measurement error—and to the conservative behavior of HTMT when a construct is defined by few, highly correlated indicators (the two depression subscales); the constructs therefore remain empirically distinct. Likewise, the difference between the observed correlation of institutional and personal-emotional adjustment (*r* = 0.17) and its model-implied value (−0.07) reflects near-zero, non-significant estimates—essentially unrelated dimensions—rather than a substantive inconsistency. Standardized loadings were all significant (*β* = 0.69–0.93; Table A1) and reliability was adequate (*ω* = 0.75–0.95; Table A3), with acceptable fit (CFI = 0.94; RMSEA = 0.074; SRMR = 0.069). The full parameter estimates—unstandardized and standardized loadings and coefficients, standard errors, *p* values, and fit indices—are reported in the Appendix under the same numbering (Supplementary Tables A1–A4), as they are not included in the main text.

## 4 Discussion

According to the scientific literature, first-year university students face serious challenges in adapting to this new academic stage (Gosai et al., 2023; Li and Lee, 2025; Samašonok et al., 2025); these difficulties can contribute to psychological distress such as fear of loneliness (Thomas et al., 2020; Wodika et al., 2024), which in turn may manifest as depressive symptomatology (Abu et al., 2023; Xie et al., 2025), with partial prior evidence from the Peruvian context (de la Cruz et al., 2023). In this sense, this study sought to evaluate the associations among these variables within an explanatory continuum.

The results show that women aligned themselves better with the demands, norms, and educational objectives of the university than men, as reflected in their higher institutional adjustment. This coincides with what has been reported in university students from the Czech Republic, where women showed better institutional adjustment (Çera et al., 2018). In the Chinese context, it has been documented that women perceive greater academic support from the university than men (Yau et al., 2013; Yau and Cheng, 2012), which offers a related framework for understanding why they integrate more favorably into institutional norms and dynamics (Martinez-Lopez et al., 2019). At the same time, women scored higher on the personal-emotional dimension; because this subscale is worded in terms of distress, this result indicates that women reported greater personal-emotional difficulties—rather than better adjustment—than men. This pattern is consistent with longitudinal evidence showing that female students tend to report higher anxiety and emotional distress, particularly during the first years of university (Gao et al., 2020). Taken together, these findings point to a dual profile: women engage more readily with the normative and institutional demands of the university, yet also experience greater emotional distress during this transition, which underscores the importance of emotional-support strategies for first-year female students rather than assuming a uniformly more favorable adjustment.

It is important to note that the relationships between the adjustment dimensions and fear of loneliness emerged more clearly in the multivariate analysis than in the bivariate one, a pattern especially notable in social adjustment, whose zero-order correlation was practically null (*r* = −0.02) but whose structural coefficient was positive and significant (*β* = 0.29). This pattern, known as cross-over suppression or net suppression (Paulhus et al., 2004), occurs when a predictor is highly correlated with other predictors in the model—in this case, social adjustment with academic (*r* = 0.67) and institutional (*r* = 0.46) adjustment—but not directly with the criterion; when estimated jointly with the other dimensions, its unique variance reveals an association different from that observed in isolation. Far from constituting an inconsistency, this finding evidences the advantage of multivariate approaches such as SEM over simple bivariate analyses, by allowing the disclosure of relationships that would otherwise remain masked by collinearity among predictors (Watson et al., 2013).

Institutional adjustment was negatively and significantly related to fear of loneliness. This finding is consistent with the Student Integration Model (Tinto, 2012), according to which the congruence between the student and the norms, values, and structure of the institution favors their retention and acts as a protective mechanism against the psychological distress derived from academic life (Rodríguez et al., 2017). In this sense, adequate institutional adjustment is linked to a decrease in the fear of loneliness, functioning as a factor associated with lower emotional isolation; students with a greater sense of institutional belonging report better psychological adjustment in the face of feelings of loneliness (Arslan, 2021).

Personal-emotional adjustment was positively associated with fear of loneliness: students reporting greater personal-emotional difficulties also reported higher levels of fear of loneliness. Far from being anomalous, this association is theoretically expected and can be understood through the affective and self-regulatory processes that operate during the transition to university. The difficulties captured by this dimension—nervousness, low mood, fatigue, and problems in managing one’s own emotions—reflect a state of heightened negative affect that increases students’ sensitivity to social threat and their tendency toward social withdrawal, thereby amplifying the apprehension of being left alone.

From the standpoint of the Cognitive Discrepancy Theory (Perlman and Peplau, 1982), this negative affect widens the perceived gap between desired and actual social relationships and heightens its aversive appraisal, so that emotional distress is translated into a stronger fear of loneliness. Consistent with this reading, emotion-regulation difficulties act as a shared vulnerability that links internalizing distress with loneliness and social apprehension (Eres et al., 2023), and longitudinal evidence indicates that emotional distress and loneliness are reciprocally and mutually reinforcing over time (Groarke et al., 2021). Accordingly, students who face greater personal-emotional difficulties during their first year are more likely both to experience and to anticipate being alone—a pattern that is also coherent with the positive association between this dimension and depression, and with evidence that better emotional adjustment tends to co-occur with lower loneliness (Labrague et al., 2021).

Academic adjustment, on the other hand, was negatively associated with fear of loneliness, indicating that students with higher levels of academic adjustment tend to present lower levels of this fear. This finding is consistent with the academic integration component proposed by Tinto (2012), according to which greater student engagement with the demands and academic system of the institution reduces the likelihood of disengagement and, thereby, the vulnerability to experiencing emotional isolation. Likewise, it supports what has been indicated by previous research regarding the inverse relationship between academic adjustment and loneliness (Mota and Ferreira, 2025; Sadoughi and Hesampour, 2016). This study constitutes, in this sense, a pioneering contribution, given that the available literature has examined loneliness in general terms but has not specifically addressed fear of loneliness.

Social adjustment, on the contrary, was positively associated with fear of loneliness, a result that diverges from what would be expected according to the social integration component of the Student Integration Model (Tinto, 2012), which posits that greater social integration should operate as a protective factor and not as a risk factor against psychological distress. This finding also contrasts with the negative relationship reported by previous studies (Franco-O’Byrne et al., 2023; Ghediya, 2026), and can be explained by the pressure that students experience during their adaptation process, which leads them to constantly compare themselves with their environment and to notice what they lack (Crone et al., 2020; Smith and Huo, 2014). In transition processes such as entering university, these comparisons tend to be upward and to deteriorate self-evaluation and well-being (McComb et al., 2023), which leads the student to experience an affective reaction such as loneliness (Arnold et al., 2021; Zell and Strickhouser, 2020). This result corroborates what has been previously indicated regarding the positive relationship between social adjustment and fear of missing out and loneliness (Boruah, 2024), and suggests that university students experience higher levels of fear of loneliness to the extent that they evaluate their social bonds as inferior to those of their peers during their adaptation process to this academic stage (Arnold et al., 2021).

Finally, fear of loneliness was significantly associated with higher levels of depression; that is, the anticipation of being alone may represent a factor of emotional vulnerability, which is similar to what was reported by Xie et al. (2025), who identified loneliness as a significant predictor of depression; this can be interpreted under the Cognitive Discrepancy Theory (Archibald et al., 1995), which explains that is theorised to underlie psychological distress. Taken together, these results show that certain facets of adaptive inadequacy can generate fear of being alone in university students undergoing this transition stage, and that this fear of loneliness may, in turn, increase the probability of depressive symptomatology, which reflects an association between fear of loneliness and depressive symptomatology in recently enrolled university students.

### 4.1 Limitations

First, being cross-sectional, it is not possible to analyze the relationships among variables over time. Second, the decision to evaluate first-order models, although methodologically justified, limits the possibility of examining interaction or mediation effects. Studies with larger samples would allow the specification of more complex integrated models. Third, the variables were assessed using self-report measures, which raises the possibility of a social desirability bias. Fourth, participants were recruited through non-probability convenience sampling at a single private, religious university—40.3% of whom shared the institution’s religious affiliation. Because a convenience sample is drawn from a conveniently accessible source rather than randomly from the target population, its findings can be generalized only to the (sub)population that shares the sample’s defining characteristics, and not to Peruvian first-year students as a whole (Andrade, 2021). The present results should therefore be read as evidence of associations within this specific student population rather than as nationally representative estimates, and replication with probabilistic, multi-institutional samples is needed before broader generalization. Fifth, potential confounding variables—such as perceived social support, personality traits, history of mental health problems, and socioeconomic status—were neither measured nor controlled for. These variables could have influenced the observed associations; future models should incorporate them. Sixth, due to the scarcity of studies specifically addressing the fear of loneliness, some of the supporting literature refers to loneliness in general. While related, the two constructs are not equivalent, which should be considered when interpreting the findings.

### 4.2 Practical implications

The first year of university constitutes the period of greatest vulnerability for the development of psychological distress, coinciding with demands for self-management, academic adjustment, and the building of new social bonds; it is therefore suggested that depression-prevention programs aimed at recently enrolled students prioritize early interventions that integrate three components: the development of emotional coping strategies, the strengthening of social support, and the reduction of perceived isolation, in line with what has been indicated by meta-analytic evidence on the reduction of loneliness (Lasgaard et al., 2026). Along these lines, peer-mentoring programs during the entry phase have been shown to simultaneously favor the academic and social integration of first-year students (Gehreke et al., 2024), in addition to producing significant reductions in depressive symptoms compared with control groups (Harra and Vargas, 2024), which makes them a strategy particularly suited to the profile of this population, which combines institutional novelty with a scarcity of consolidated support networks.

Complementarily, onboarding programs focused on strengthening social and personal competencies from the first day of classes have been shown to be effective in sustaining the well-being of first-year students in the face of the demands inherent to this transition (Limarutti et al., 2021), so their joint implementation with mentoring strategies could constitute a comprehensive and cost-effective approach to preventing the development of depressive symptomatology associated with fear of loneliness at this critical stage of university life.

## 5 Conclusion

Overall, the findings indicate that the transition to university life may erode the adaptive capacities of first-year students, and that these difficulties are associated both with fear of loneliness, arising from the new environment, and with depressive symptomatology linked to the emotional strain of this stage. Accordingly, it is recommended that educational authorities prioritize attention to these issues among recently enrolled students.

## Data Availability

The datasets generated and analyzed for this study can be found in the Open Science Framework (OSF) repository: https://doi.org/10.17605/OSF.IO/J2WN6.
