# Supplementary material for: Adaptation to university life, fear of loneliness, and depression in first-year students at a private university in the Peruvian Amazon: a structural equation model
Source: Front Psychol. 2026 Oct 14;17:1935089. doi: 10.3389/fpsyg.2026.1935089 (PMC13713792; doi:10.3389/fpsyg.2026.1935089)
Supplement: Supplementary file 1 [file Supplementary_file_1.docx]

Supplementary Material

# 1 Supplementary Data

The datasets generated and analyzed for this study are available in the Open Science Framework (OSF) repository: [**https://doi.org/10.17605/OSF.IO/J2WN6**](https://doi.org/10.17605/OSF.IO/J2WN6)

# 2 Supplementary Figures and Tables

## 2.1 Supplementary Figures

**Supplementary Figure A1.** Complete structural equation model, including all observed indicators of each latent variable, with standardized factor loadings, structural coefficients, and covariances.

## 2.2 Supplementary Tables

**Supplementary Table A1.** Parameters of the structural equation model

| Factor | Indicator | B | SE | β | z | p |
| --- | --- | --- | --- | --- | --- | --- |
| Institutional adj. | SACQ1 | 1.000 | — | .839 | — | — |
|  | SACQ2 | 1.046 | 0.036 | .877 | 28.9 | < .001 |
|  | SACQ3 | 1.006 | 0.038 | .844 | 26.4 | < .001 |
|  | SACQ4 | 1.061 | 0.036 | .890 | 29.5 | < .001 |
|  | SACQ5 | −0.710 | 0.059 | −.596 | −12.1 | < .001 |
|  | SACQ6 | −0.737 | 0.060 | −.618 | −12.3 | < .001 |
| Personal-emotional | SACQ7 | 1.000 | — | .728 | — | — |
|  | SACQ8 | 0.956 | 0.034 | .696 | 27.8 | < .001 |
|  | SACQ9 | 1.099 | 0.036 | .800 | 30.5 | < .001 |
|  | SACQ10 | 1.109 | 0.038 | .808 | 29.1 | < .001 |
|  | SACQ11 | 1.091 | 0.038 | .794 | 28.4 | < .001 |
|  | SACQ12 | 1.182 | 0.041 | .861 | 29.0 | < .001 |
|  | SACQ13 | 1.106 | 0.039 | .805 | 28.7 | < .001 |
|  | SACQ14 | 1.016 | 0.039 | .740 | 26.1 | < .001 |
|  | SACQ15 | 0.945 | 0.043 | .688 | 21.9 | < .001 |
|  | SACQ16 | 1.081 | 0.042 | .787 | 25.5 | < .001 |
|  | SACQ17 | 1.092 | 0.041 | .795 | 26.5 | < .001 |
|  | SACQ18 | 1.197 | 0.041 | .871 | 29.0 | < .001 |
|  | SACQ19 | 1.140 | 0.037 | .830 | 30.5 | < .001 |
| Academic adj. | SACQ20 | 1.000 | — | .827 | — | — |
|  | SACQ21 | 0.972 | 0.028 | .804 | 34.2 | < .001 |
|  | SACQ22 | 0.995 | 0.026 | .823 | 38.2 | < .001 |
|  | SACQ23 | 1.045 | 0.024 | .865 | 43.8 | < .001 |
| Social adj. | SACQ24 | 1.000 | — | .921 | — | — |
|  | SACQ25 | 0.886 | 0.021 | .816 | 41.4 | < .001 |
|  | SACQ26 | 0.834 | 0.025 | .769 | 33.6 | < .001 |
|  | SACQ27 | 0.954 | 0.018 | .879 | 53.1 | < .001 |
| Fear of loneliness | BSFL1 | 1.000 | — | .788 | — | — |
|  | BSFL2 | 1.184 | 0.034 | .932 | 35.1 | < .001 |
|  | BSFL3 | 1.163 | 0.034 | .916 | 34.1 | < .001 |
|  | BSFL4 | 0.970 | 0.038 | .764 | 25.7 | < .001 |
|  | BSFL5 | 1.045 | 0.034 | .823 | 30.9 | < .001 |
| Depression | Somatic | 1.000 | — | .795 | — | — |
|  | Cognitive-affective | 2.205 | 0.166 | .910 | 13.2 | < .001 |

**Supplementary Table A2.** Structural model (regression coefficients)

| Structural path | B | SE | β | z | p |
| --- | --- | --- | --- | --- | --- |
| Institutional adj. → Fear of loneliness | −0.206 | 0.064 | −.220 | −3.22 | .001 |
| Personal-emotional → Fear of loneliness | 0.531 | 0.054 | .491 | 9.93 | < .001 |
| Academic adj. → Fear of loneliness | −0.319 | 0.093 | −.335 | −3.43 | < .001 |
| Social adj. → Fear of loneliness | 0.245 | 0.081 | .286 | 3.01 | .003 |
| Fear of loneliness → Depression | 1.961 | 0.185 | .806 | 10.58 | < .001 |

**Supplementary Table A3.** Reliability and average variance extracted

| Construct | α | ω | AVE |
| --- | --- | --- | --- |
| Institutional adjustment | .70 | .75 | .62 |
| Personal-emotional | .94 | .95 | .62 |
| Academic adjustment | .87 | .88 | .69 |
| Social adjustment | .88 | .89 | .72 |
| Fear of loneliness | .91 | .91 | .72 |
| Depression | .74 | .87 | .79 |

**Supplementary Table A4.** Discriminant validity (HTMT)

| Construct | 1 | 2 | 3 | 4 | 5 | 6 | AVE |
| --- | --- | --- | --- | --- | --- | --- | --- |
| 1. Institutional adjustment | 1.00 |  |  |  |  |  | .62 |
| 2. Personal-emotional | .09 | 1.00 |  |  |  |  | .62 |
| 3. Academic adjustment | .60 | .12 | 1.00 |  |  |  | .69 |
| 4. Social adjustment | .61 | .07 | .79 | 1.00 |  |  | .72 |
| 5. Fear of loneliness | .28 | .41 | .11 | .08 | 1.00 |  | .72 |
| 6. Depression | .15 | .57 | .17 | .11 | .58 | 1.00 | .79 |
